# Study on DNA Storage Encoding Based IAOA under Innovation Constraints

**DOI:** 10.3390/cimb45040233

**Published:** 2023-04-18

**Authors:** Haigui Du, Shihua Zhou, WeiQi Yan, Sijie Wang

**Affiliations:** 1 Key Laboratory of Advanced Design and Intelligent Computing, Ministry of Education, School of Software Engineering, Dalian University, Dalian 116622, China; 2 School of Engineering, Computer and Mathematical Sciences, Auckland University of Technology, Auckland 1010, New Zealand

**Keywords:** DNA storage, DNA encoding, arithmetic optimization algorithm, double-matching constraint, error-pairing constraint

## Abstract

With the informationization of social processes, the amount of related data has greatly increased, making traditional storage media unable to meet the current requirements for data storage. Due to its advantages of a high storage capacity and persistence, deoxyribonucleic acid (DNA) has been considered the most prospective storage media to solve the data storage problem. Synthesis is an important process for DNA storage, and low-quality DNA coding can increase errors during sequencing, which can affect the storage efficiency. To reduce errors caused by the poor stability of DNA sequences during storage, this paper proposes a method that uses the double-matching and error-pairing constraints to improve the quality of the DNA coding set. First, the double-matching and error-pairing constraints are defined to solve problems of sequences with self-complementary reactions in the solution that are prone to mismatch at the 3′ end. In addition, two strategies are introduced in the arithmetic optimization algorithm, including a random perturbation of the elementary function and a double adaptive weighting strategy. An improved arithmetic optimization algorithm (IAOA) is proposed to construct DNA coding sets. The experimental results of the IAOA on 13 benchmark functions show a significant improvement in its exploration and development capabilities over the existing algorithms. Moreover, the IAOA is used in the DNA encoding design under both traditional and new constraints. The DNA coding sets are tested to estimate their quality regarding the number of hairpins and melting temperature. The DNA storage coding sets constructed in this study are improved by 77.7% at the lower boundary compared to existing algorithms. The DNA sequences in the storage sets show a reduction of 9.7–84.1% in the melting temperature variance, and the hairpin structure ratio is reduced by 2.1–80%. The results indicate that the stability of the DNA coding sets is improved under the two proposed constraints compared to traditional constraints.

## 1. Introduction

From ancient stone inscriptions to current digital storage methods, such as USB flash drives and hard drives, storage is an indispensable way to preserve and record data. However, with global informatization, the proliferation of the data volume has posed difficulties for data storage. By 2025, the global data volume is expected to grow to 175 ZB [1], which is equivalent to 5.03 × 10^5^ PE per day. The existing storage media will not be able to meet such a demand for data storage. Conventional storage media, such as CDs and floppy disks, have problems of high cost, short storage times, and difficulties in the data storing process [2]. Thus, there is an urgent need for the development of new storage media to solve these problems. Molecules of nucleic acids were first studied by Watson et al. [3] in 1953, and then, life forms were analyzed from the perspective of molecular biology, which opened up new horizons in research on biogenetics. Deoxyribonucleic acid (DNA) is a storage medium endowed by nature. Compared with conventional storage media, DNA storage has the advantages of a large storage capacity, long storable time, and easy storage, and thus, it has become a widely used storage tool [4]. Each gram of DNA can store 215 PE of data [5], and it takes only 2.34 × 10^3^ g of DNA to store 5.03 × 10^5^ PE of data, which is equivalent to 5.15 million 100 TB capacity hard drives. In addition, DNA is extremely stable and can survive for thousands of years under harsh conditions, thus far exceeding the survival time of silicon devices [4]. Since its discovery, DNA storage technology has been continuously applied to solve various problems in many fields, including archival data storage, data encryption, and DNA computing.

In 2012, Church and Kosuri et al. [6] successfully encoded and decoded digital information using next-generation DNA synthesis and sequencing techniques. Goldman et al. [7] stored a certain amount of data in DNA, solving the problem of previous DNA storage methods, which can encode only a small amount of information, and showed that the original data could be restored with perfect accuracy. In 2015, a DNA storage architecture proposed by Yazdi et al. [8] implemented random data reads and re-edited the stored data at all locations within the data blocks. Further, in 2017, Erlich et al. [5] introduced a data storage method known as the DNA fountain, where DNA oligonucleotides store data with a total length of 2.14 × 10^6^ bytes, including a complete computer system, film and television productions, and other files, and the data can be successfully decoded. In 2019, Benner et al. [9] discovered four additional nucleotides, creating a breakthrough DNA molecule with eight letters. In the same year, Ping et al. [10] proposed a codec system called Yin-Yang that could successfully store files of different formats in a pool of synthetic DNA oligonucleotides. Choi et al. [11] proposed the use of simply merged bases as coding characters and experimentally obtained 3.37 bits/character, which more than doubled the original maximum information capacity. Anavy et al. [12] encoded 6.4 MB as composite DNA, using only 80% of the original synthesis cycle and distinguishable compositional media. In 2020, Zhang and Kong et al. [13] proposed an improved Base64 method and obtained a storage ratio of 1.77 bits/nucleotide in a single strand of DNA. Ping et al. [14] developed Chamaeleo, which is a scalable integration and evaluation platform for DNA storage codec methods, to perform a systematic quantitative analysis and evaluate existing codec methods in a modular integration manner. This platform supports the output of codec methods consisting of different types of files in a meritocratic manner. Cao and Li et al. [15,16] proposed new constraints for constructing DNA storage codebooks, which could effectively improve the quantity and quality of DNA storage sets. In 2021, Shomorony et al. [17] analyzed the DNA storage systems using a noisy shuffle sampling channel model. They showed that an index-based coding scheme could achieve the best results in many scenarios based on specific noise and sampling assumptions. According to Li et al. [18], this DNA storage system could self-interpret its stored data without using any external tools. Li et al. designed an index for random access to the stored files. For DNA storage systems, Schwarz et al. [19] proposed a software framework called NOREC4DNA, which can be used to test and improve near-optimal rate-free erasure codes (NORECs). Park et al. [20] presented a planning strategy that used a ravenous calculation to reduce the typical piece error rate by 2.3455 pieces brought about by a nucleotide blunder, which represented a reduction of 20.5% compared to the random mapping method. Zheng et al. and Wu et al. [21,22] developed methods for constructing well-performing DNA coding sets and introduced new constraints to construct more robust coding sets. In 2022, Li et al. [23] proposed an efficient method for constructing DNA coding sets, which can effectively improve the quantity and quality of DNA coding sets. Ren et al. [24] developed two highly reliable coding systems, named the RALR and the RABR, for four, six, and eight nucleotides. The typical coding steps of the RALR framework using four, six, and eight nucleotides obtained 1.27 bits/nucleotide, 1.61 bits/nucleotide, and 1.85 bits/nucleotide in the error-correctable and uncompressed cases, respectively. In contrast, the average coding rates of RABR systems using four, six, and eight nucleotides were 1.50 bits/nucleotide, 2.00 bits/nucleotide, and 2.35 bits/nucleotide, respectively.

To reduce the occurrence of non-specific hybridization reactions and thus the chance of errors in the DNA storage process, rational coding of DNA sequences is required. The DNA sequences are subject to their base complementary pairing reactions in solution, and this study used a double-matching constraint to reduce the probability of this situation occurring. The 3′ end is the focus of amplification, and its pairing affects the enzyme extension and determines the specificity and efficiency of synthesis. Only by strict restriction of the bases at the 3′ end can the desired amplification product be obtained. Therefore, this study introduced an error-pairing constraint to achieve such a restriction. In addition, this study improved the arithmetic optimization algorithm (AOA) and proposed the IAOA, which was combined with traditional constraints to construct DNA coding sets that comply with specific constraints and restrictions. The lower bounds of these coding sets were improved, but there were still undesirable secondary structures that affect the stability of DNA sequences. Therefore, to obtain high-quality DNA coding sets, this paper used the IAOA to construct coding sets under double-matching and error-pairing constraints. The effectiveness of the constraints and the quality of the coding set were evaluated by tests such as the concentration before and after entering the solution, the number of hairpin structures, and the melting temperature. The results show that the two proposed constraints can effectively improve the quality of the DNA coding sets.

## 2. Algorithm Description

### 2.1. Arithmetic Optimization Algorithm

In 2021, Abualigah et al. [25] proposed the AOA and used it to solve practical problems. The AOA uses four arithmetic operators: multiplication, division, subtraction, and addition.

The AOA search process can be divided into two main phases: the exploration phase and the development phase. Before the AOA starts the optimization process, it judges and selects the search phase (exploration or development). The arithmetic optimization acceleration function (Math Optimizer Accelerated) is an important factor that is used to control the exploration and development phases, and it is given by Equation (1). The AOA sets a random number $r_{1}$ after updating the MOA. If $r_{1}>MOA$, the AOA executes the multiplication and division arithmetic operations (executes the exploration phase); otherwise, it executes the addition and subtraction arithmetic operations (executes the development phase).

(1) $$MOA\left(t\right)=(\frac{max-min}{T})\times t+min$$

The minimum and maximum values of MOA are denoted by min and max, respectively, and they are set to 0.2 and 1, respectively; t and T denote the current and maximum number of iterations of the algorithm, respectively.

The exploration phase of the AOA focuses on random exploration over regions using two operators: the division operator and the multiplication operator. The development phase uses more concentrated addition and subtraction operators to explore several specified regions deeply to find an optimal solution. The location update equations of the two phases are expressed by Equations (2) and (4).

(2) $$x_{i,j}\left(t+1\right)=\left\{\begin{array}{c} best\left(x_{j}\right)÷\left(MOP+\varepsilon \right)\times V_{j}\;r2\leq 0.5\\ best\left(x_{j}\right)\times MOP\times V_{j}\;r2>0.5 \end{array}\right.$$

(3) $$V_{j}=\left({UB}_{j}-{LB}_{j}\right)\times \mu +{LB}_{j}$$

(4) $$x_{i,j}\left(t+1\right)=\left\{\begin{array}{c} best\left(x_{j}\right)-MOP\times V_{j}\;r3\leq 0.5\\ best\left(x_{j}\right)+MOP\times V_{j}\;r3>0.5 \end{array}\right.$$

where $V_{j}$ is defined by Equation (3); $x_{i,j}\left(t\right)$ is the *j*th position of the *i*th solution in the current iteration;$\;best(x_{j})$ is the *j*th position of the optimal solution in the current iteration; ε is defined as an integer; ${UB}_{j}$ and ${LB}_{j}$ denote the minimum and maximum values of the *j*th position, respectively; μ is a parameter that regulates the search phase of AOA, and it was set to 0.5 in [25]; MOP is the mathematical optimizer probability, which is an important coefficient affecting the position update, and it is given by Equation (5); and α is a sensitive parameter whose value is set to 5, and it defines the development accuracy of the iterative process of AOA.

(5) $$MOP\left(t\right)=1-\frac{t^{1/\alpha }}{T^{1/\alpha }}$$

### 2.2. IAOA

Due to the advantages of having few parameters and a fast execution efficiency, the AOA has been widely used to solve many practical problems, such as engineering optimization and path planning. For instance, in [26], an improved adaptive arithmetic optimization algorithm using a dual-parallel communication technique was proposed and applied to solve the robot path planning problems. An improved arithmetic optimization approach for a mechanical engineering design was proposed in [27] and used with natural logarithms and exponential operators. In [28], an arithmetic optimization algorithm based on perturbations of primitive functions was proposed. By adding the perturbations produced by six primitive functions to each of the significant parameters, this algorithm can improve its performance in solving the economic load scheduling problem. Further, in [29], the authors proposed a method based on an evolutionary optimization algorithm and applied it to solve the multilevel thresholding problem, generating better solutions and segmented images.

The arithmetic optimization algorithm proposed in the literature has improved the solution accuracy and algorithm stability to a certain extent. However, the AOA can easily fall into a local optimum and slowly converge. This study proposes the IAOA based on two strategies to overcome these two drawbacks. In the IAOA, the perturbations generated by the elementary functions are added to MOA and MOP, respectively, and double adaptive weights are added to the position update formula. This enables the algorithm to perform an adequate global search, avoid falling into locally optimal solutions, and improve the convergence speed of AOA and the accuracy of the obtained solutions.

#### 2.2.1. Elementary Function Perturbation

The AOA has two important factors, MOA and MOP, which greatly affect the exploration and development capability of the AOA. As the number of iterations increases, the original MOA increases, while the original MOP decreases. In this study, random perturbations generated by the primitive function are introduced to improve the two parameters. This can balance the exploration and exploitation performance of the algorithm, reduce the likelihood of the algorithm falling into a local optimum, and improve the convergence ability of the algorithm [28].

Further, the parameters MOP and MOA are multiplied by the coefficients g and h, respectively, to improve the parameters using the random fluctuations generated by the elementary function; the MOP and MOA are expressed by Equations (6) and (7), respectively.

(6) $$MOP\left(t\right)=\left(1-\frac{t^{\frac{1}{\alpha }}}{T^{\frac{1}{\alpha }}}\right)\times g$$

(7) $$MOA\left(t\right)=\left(min+\left(max-min\right)\times \frac{t^{1/3}}{T^{1/3}}\right)\times h$$

(8) $$g=\left|m_{1}\times acos(m_{3})\right|$$

(9) $$h=\left|m_{2}\times acos(m_{3})\right|$$

The values of the coefficients g and h are calculated using Equations (8) and (9); $m_{1}$ and $m_{2}$ are both constant parameters, $m_{1}$ is set to 1.2, $m_{2}$ is set to 0.65, and $m_{3}$ is a random number between zero and one.

#### 2.2.2. Double Adaptive Weighting Strategy

In the AOA, the globally explored and locally developed position update formulas are crucial in the search for an optimal solution. However, some solutions may fall into the local optimum because of the limited search area. In this study, a dual adaptive weighting strategy was introduced. The weight curve gradually reduces according to the level at which the algorithm falls into a local optimum [30]. Adaptive weight $w_{1}$ is used when the AOA performs division and subtraction operators, and adaptive weight $w_{2}$ is used when it performs multiplication and addition operators. This strategy avoids local optimal stagnation while improving the local search capability of IAOA and the accuracy of the solutions. The position update formulas for the two stages are given by Equations (10) and (11).

(10) $$x_{i,j}\left(t+1\right)=\left\{\begin{array}{c} w_{1}\times best\left(x_{j}\right)÷\left(MOP+\varepsilon \right)\times V_{j}\;\;r2\leq 0.5\\ w_{2}\times best\left(x_{j}\right)\times MOP\times V_{j}\;\;r2>0.5 \end{array}\right.$$

(11) $$x_{i,j}\left(t+1\right)=\left\{\begin{array}{c} w_{1}\times best\left(x_{j}\right)-MOP\times V_{j}\;\;r3\leq 0.5\\ w_{2}\times best\left(x_{j}\right)+MOP\times V_{j}\;\;r3>0.5 \end{array}\right.$$

(12) $$w_{1}={\left(1-\frac{t}{T}\right)}^{1-\tan\left(\pi \times \left(rand-0.5\right)\right)\times \frac{S}{T}}$$

(13) $$w_{2}={\left(2-\frac{2t}{T}\right)}^{1-\tan\left(\pi \times \left(rand-0.5\right)\right)\times \frac{S}{T}}$$

The weight values $w_{1}$ and $w_{2}$ are given by Equations (12) and (13), where the current and maximum numbers of iterations are denoted by t and T, respectively; rand is a random number in the range between zero and one. The pseudo-code of the IAOA is shown in Algorithm 1.


**Algorithm 1. Pseudo-code of the IAOA.**
1: Initialization parameters and population location X_i_ (i = 1,2...N)
2: While(t < T)
3: $MOP\left(t\right)=\left(1-\frac{t^{\frac{1}{\alpha }}}{T^{\frac{1}{\alpha }}}\right)\times g,MOA\left(t\right)=\left(min+\left(max-min\right)\times \frac{t^{1/3}}{T^{1/3}}\right)\times h$
4: for i = 1 : N
5:  for j = 1 : N
6:   if r1 > MOA
7:    if r2 > 0.5 (exploration phase)
8:     $x_{i,j}\left(t+1\right)=w_{1}\times best\left(x_{j}\right)÷\left(MOP+\varepsilon \right)\times V_{j}$
9:    else
10:     $x_{i,j}\left(t+1\right)=w_{2}\times best\left(x_{j}\right)\times MOP\times V_{j}$
11:    end if
12:    if r3 > 0.5 (development phase)
13:     ${x_{i,j}\left(t+1\right)=w}_{1}\times best\left(x_{j}\right)-MOP\times V_{j}$
14:    else
15:     $x_{i,j}\left(t+1\right)=w_{2}\times best\left(x_{j}\right)+MOP\times V_{j}$
16:    end if
17:   end if
18:  end for
19: end for
20: end while
21: Return to the optimal solution

It shows how the IAOA finds an optimal solution.

### 2.3. Benchmark Function Comparison

To illustrate the performance of the IAOA better, 13 classical benchmark functions were used as evaluation criteria [31], where F1–F7 denote single-peaked functions and F8–F13 are multipeaked functions. Meanwhile, the original AOA [25] and several representative metaheuristic algorithms, including the multiverse algorithm (MVO) [32], the gravitational search algorithm (GSA) [33], the whale optimization algorithm (WOA) [34], the salp swarm algorithm (SSA) [35], the sine cosine algorithm (SCA) [36], the EAOA using only the primary function perturbation strategy, the DAOA using only the adaptive weighting strategy, and the pAOA [28], were compared with the IAOA. The performance results of the comparison algorithms were obtained from the studies of Hao et al. [28] and Zheng et al. [37]. In addition, the algorithm tests were performed in the same experimental environment. The maximum number of iterations, population size, and test dimension were set to 500, 30, and 30, respectively. By calculating the minimum, mean, and standard deviation values and executing each function 30 times independently, the randomness of the algorithm results was reduced. A lesser mean value and a lesser standard deviation value were associated with a better and steadier algorithm performance while solving the algorithm minimum problem. Thus, this study assessed the performance and stability of the algorithm based on the average value (AVE) and the standard deviation value (STD). The results are presented in Table 1 and Table 2, where the best results are presented in bold.

Because a single-peaked function only has a globally optimal solution, the development capability of optimization algorithms has usually been evaluated using the single-peaked functions F1–F7 [37]. They contain nice functions as well as malicious cases causing poor or slow convergence to a single global extremum [31]. As shown in Table 1, the IAOA achieved better results than the other metaheuristics; particularly, for the functions F1–F4, the mean and variance of the IAOA reached the global optimum, but the functions F6 and F7 did not reach the global optimum; this may be due to the large optimization interval of the functions, for which IAOA did not converge well in the early stage. However, the average values of functions F6 and F7 were improved compared to the original AOA. The mean values of four of the seven single-peaked test functions converge to zero, indicating that the IAOA converged to the global optimum in theory. Additionally, the variances were zero, indicating that the algorithm was highly stable. Compared with other optimization algorithms, the IAOA achieved a significant advantage in the development stage.

The exploratory power of the IAOA was evaluated using multipeaked functions F8–F13. The test functions contain a high number of locally optimal solutions, and the number of local optimal solutions increases exponentially as the number of dimensions in the problems increased. This adds difficulty to the solution. Therefore, they could provide reliable insights into the exploration ability of the IAOA. The AVE and STD of functions F9–F11 of the IAOA reached optimal values compared to the other algorithms. The test results of functions F8 and F13 of the IAOA were not the best compared to other algorithms; this may be due to the large exploration space of the functions, which our algorithm did not explore fully at a later stage. However, the mean values of these functions of the IAOA were better than those of the original AOA. The test results showed that the IAOA had a stronger exploration capability than the other algorithms.

**Figure 1:**
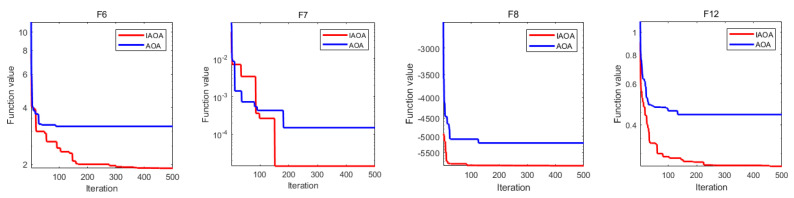
Convergence curves of functions F6, F7, F8, and F12. The blue and red curves represent the convergence curves of the AOA and IAOA, respectively; the horizontal axis denotes the number of iterations, where the maximum number of iterations is 500; the vertical axis presents the optimal value of the objective function.

To demonstrate the performance of IAOA more clearly, the convergence iteration curves of several test functions were chosen for comparison. To evaluate the general optimality of IAOA and enhance the persuasive power of data, the functions that did not reach optimal values were selected, including functions F6, F7, F8, and F12. Figure 1 shows that functions F6 and F7 did not fall into the original local optimum and converged to the global optimum after jumping out, while functions F8 and F12 rapidly converged to the optimal solution. The IAOA further improved the convergence speed and optimization capability compared to the original algorithm.

### 2.4. Wilcoxon Rank Sum Test

To assess the variability of the algorithm test results, this study performed a non-parametric Wilcoxon rank sum test [38], and the results are presented in Table 3. In the Wilcoxon test, the IAOA was used as a control algorithm. The obtained *p*-values were used to evaluate the test results; when p<0.05, there was a significant difference between the two algorithms. According to the results in Table 3, for all seven pairs of algorithm comparisons, the *p*-value was less than 0.05, indicating a significant difference between the IAOA and the other seven algorithms. This demonstrates that the proposed algorithm is highly competitive.

## 3. Construct DNA Storage Sets

Due to the diversity of DNA, nonspecific hybridization reactions may occur in DNA sequences. To avoid nonspecific hybridization reactions of DNA sequences during data storage, it is necessary to impose strict constraints on the DNA sequence design. Strict constraints are essential to construct high-quality DNA storage sets, which can enhance sequence robustness and reduce errors during DNA sequencing. The constraints used in sequence design are presented in the following text.

### 3.1. Double-Matching Constraint

To avoid undesired complementary reactions of DNA sequences in solution, a double-matching constraint is proposed in this paper. The simulation software NUPACK was used to design this constraint. NUPACK software is used by researchers in the life sciences to analyze and design nucleic acid structures. The DNA sequence is entered into NUPACK, and the status of the DNA sequence in solution is assessed by observing its change. In the NUPACK evaluation, ideally, DNA sequences should not react with each other or undergo their own base complementary pairing reactions, thus maintaining the maximum concentration in the solution. If the complementary base pairs in the solution react, the concentration of DNA sequences will be reduced, thus affecting the quality of the DNA storage sets [15]. The simulation evaluation result of sequences introduced into NUPACK is obtained by $c=C_{output}/C_{input}$, where $C_{input}$ and $C_{output}$ denote the DNA sequence concentrations before and after entering the solution, respectively. Thus, the DNA sequence quality can be evaluated based on the c-value; a large c-value indicates high quality.

**Figure 2:**
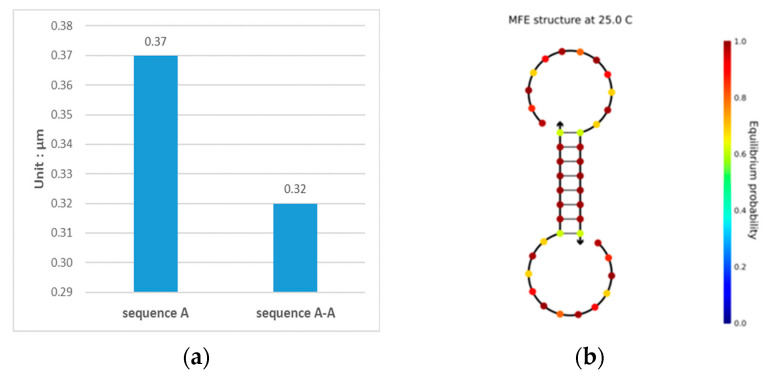
Analysis results of sequence TCTGTACTGCTGACTCGAGT. (**a**) Sequence species and concentration after the sequence enters the solution; sequences A and A-A obtained by the complementary pairing reaction after the analyzed sequence enters the solution; (**b**) the secondary structure diagram of sequence A-A.

When a DNA sequence is evaluated using NUPACK, a self-complementary reaction may occur when a DNA sequence is added to the solution, as shown in Figure 2. The sequence shown in Figure 2 was taken from the study of Chaves-González et al. [39], and it was obtained when a 1-μm sequence was fed to the NUPACK input. Figure 2a shows that there is a structural sequence A-A in the solution other than sequence A, and at this point, $C_{output}=0.37\;\mu m$, $C_{input}=1\;\mu m$ and c=37%. The concentration of sequence A in the solution is reduced, indicating that the quality of the sequence has decreased. Thus, the chance of errors in the reaction during DNA storage increases. The structure of sequence A-A is shown in Figure 2b, where it can be seen that the underlined six bases undergo complementary pairing reactions in the solution, forming a stable pairing structure. The pairing structure is not expected, and it makes the original sequence less concentrated, while the c-value decreases due to the presence of a stable and unavailable structure.

**Figure 3:**
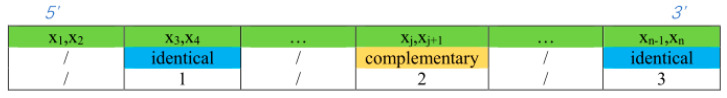
Double-matching constraint. The bases are connected according to the 5′ -> 3′ end arrangement. The first line of data in the figure denotes sequence x with a base number n; two consecutive bases are a combination, divided into n/2 base combinations, such as $\left(x_{1},x_{2}\right)$, assuming that $\left(x_{1},x_{2}\right)$ is the original base combination and looking for the base combination associated with it in the sequence. The second row of data indicates the relationships between the current base combination and $\left(x_{1},x_{2}\right)$; “identical” means the two combinations are identical, and “complementary” means the two combinations are complementary. The third row of data denotes the number of combinations in the sequence that are identical or complementary to $\left(x_{1},x_{2}\right)$, that is, the count in Equation (14). There are four combinations that are related, including the combination $\left(x_{1},x_{2}\right)$. Therefore, the bases of one of the combinations $\left(x_{3},x_{4}\right)$, $\left(x_{j},x_{j+1}\right)$, and $\left(x_{n-1},x_{n}\right)$ are selected for replacement, and then it is re-judged count>3; if so, then the base replacement continues; otherwise, the next base combination is selected as the original base combination, scanning continues, and the above steps are repeated.

**Figure 4:**
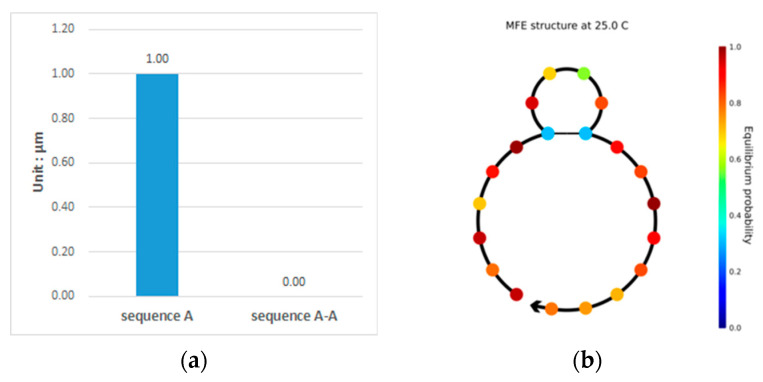
Analysis of the sequence TCTGTACTGCTGACTCGGGC. (**a**) Sequence species and concentration present after the sequence enters the solution, including sequence A and sequence A-A obtained by the complementary pairing reaction after it enters the solution; (**b**) shows the secondary structure diagram of sequence A.

This paper proposes a double-matching constraint to reduce the generation of such unavailable structures, as shown in Figure 3. This constraint combines two consecutive bases and judges whether more than three of the combinations are complementary or identical base pairs. In the case where more than three combinations are perfectly complementary or identical, two different bases of one of the combinations are selected for the exchange. If the selected bases are complementary, the noncomplementary bases are selected for replacement. The results of the exchange are shown in Figure 4. After the double-matching constraint exchange has been completed, the sequence TCTGTACTGCTGACTCGGGC is obtained, and c=100%, as shown in Figure 4a. The indicator in the NUPACK shows that the concentration of sequence A is 1 μm, and the concentration of sequence A-A is zero. The six bases in the sequence neither undergo complementary pairing reactions nor produce undesired pairing structures; thus, the sequence in the solution maintains its original state, which is the expected ideal state. Thus, the proposed constraint can effectively increase the concentration of sequence A involved in the reaction while reducing the error rate in the DNA sequence hybridization reaction [16]. The structure of sequence A is shown in Figure 4b, where the color of the paired bases changes; when the color tends toward the lower center of the right color bar, sequence A is likely to form an unstable available structure. The formula for selecting bases for exchange is given in Equation (14).

In Equation (14), x is a DNA sequence, n denotes the total number of bases in the sequence, $x^{\prime }$ is a subsequence of x, and count is the number of subsequences identical or complementary to $x^{\prime }$, satisfying the condition $x^{\prime }=(x_{j},x_{j+1})$, j∈[1,n−1]. The double-matching constraint is expressed as follows:

(14) $$f_{double}\left(x\right)=\left\{\begin{array}{c} f_{double}\left(x^{\prime }\right)\;count>3\\ x^{\prime }\;count\leq 3 \end{array}\right.$$

### 3.2. Error-Pairing Constraint

When a sequence is stored as a double strand, the A-T and G-C base pairs are connected by two and three hydrogen bonds, respectively, where the G-C base pairs are steadier than A-T base pairs [22]. The key to amplification is the 3′ end, whose pairing affects the enzyme extension and defines the specificity and efficiency of synthesis. The binding of the primer and template can be made more stable by using particular numbers of G and C bases. If the distribution of bases at the 3′ end is not reasonable, a mismatch situation may be caused. In the case of a mismatch, the synthesis of the strand can be triggered, and a nonideal amplification product can be obtained. In the primer design notes, it is stated that when the final base of the primer is A, strand synthesis can be initiated, even in the case of mismatches, but when it is T, the efficiency of mismatch initiation is significantly reduced [40].

**Figure 5:**
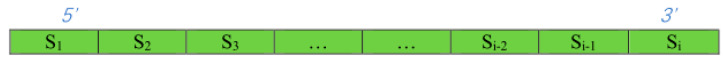
Error-pairing constraint. The figure shows sequence S of i bases, where the bases are connected according to the 5′ -> 3′ end arrangement, and this constraint is mainly enforced by judging the last base $S_{i}$. When $S_{i}$ is base G or C, the mismatch level (MPL) is set to one; when $S_{i}$ is base T, the MPL is set to two; and finally, when $S_{i}$ is base A, the MPL is set to three.

To ensure the 3′ end of the sequence can be strictly paired and stabilized, this study introduces an error-pairing constraint to restrict the distribution of bases at the 3′ end. The proposed error-pairing constraint is shown in Figure 5. The error-pairing constraint in the DNA sequence amplification process indicates the possibility of mismatches at the 3′ end and the efficiency degree of mismatch initiation, which is expressed by MPL. The greater the value of *MPL* is, the higher the mismatch occurrence possibility and the mismatch initiation efficiency are. In sequence S, the rating number is one, two, or three when the last base is G (or C), T, or A, respectively. The error-pairing constraint is given in Equation (15).


(15) $$S=S_{1}S_{2}\ldots S_{i-1}S_{i},\;S_{i}=\left\{\begin{array}{c} G/C\;MPL=1\\ T\;MPL=2\\ A\;MPL=3 \end{array}\right.$$

### 3.3. Hamming Distance Constraint

The Hamming distance constraint [41] of sequences x and y with a length of *n* satisfies the condition $D\left(x,y\right)\geq d$, where $D\left(x,y\right)$ represents the number of positions in sequence x that are different from the *i*th position in sequence y, i∈[1,n]. The Hamming distance ($D\left(x,y\right)$) is given by

(16) $$D\left(x,y\right)=\sum_{i=1}^{n}h\left(x_{i},y_{i}\right),h\left(x_{j},y_{j}\right)=\left\{\begin{array}{c} 0,\;x_{i}=y_{i}\\ 1,\;x_{j}\neq y_{j} \end{array}\right.$$

### 3.4. GC Content Constraint

GC content [42] represents the ratio of the number of bases C and G to the total number of bases in a DNA sequence. The GC content constraint is an indispensable DNA sequence that can directly influence the performance of DNA sequence and is generally set to approximately 50% to ensure robustness against errors. It is assumed that, in a DNA sequence, the total number of bases is n, the number of bases G is denoted by x, and the number of bases C is denoted by y. Then, the GC content represents the percentage of the ratio of the sum of *x* plus *y* to *n*. The GC content (c) of a sequence is given by

(17) $$c=\frac{x+y}{n}\times 100\%$$

### 3.5. No-Runlength Constraint

The no-runlength constraint [5] requires DNA sequences to not contain consecutive repeating bases, and long runs of identical nucleotides can prompt errors in DNA coding. For instance, in the sequence TAAAACG, since base A is repeated consecutively, the repeating base TAAAACG could be misread as TAAACG or TAACG during sequencing, leading to the loss of DNA information. Meanwhile, contiguous bases may lead to decoding errors during the decoding process, resulting in uncontrollable hybridization reactions throughout the sequence. Therefore, the no-runlength constraint is used to prevent the occurrence of consecutive identical bases. The no-runlength constraint is given by

(18) $$x_{i}\neq x_{i+1}\;\;i\in \left[1\right.,\left.n-1\right]$$

### 3.6. DNA Storage Sets Constructed Using Traditional Combinatorial Constraints

In this study, the IAOA is used to construct DNA storage coding sets to improve the lower bound and quality of storage sets. The results of the entire simulation experiment are run on a desktop computer with an Intel Core i5 2.30-GHZ processor and 8 GB storage space, and MATLAB R2018b is used.

The specific steps are as follows:

Step 1: Initialize algorithm parameters, generate sets of candidate solutions satisfying the combinatorial constraints, and integrate them into the initial sequence sets;

Step 2: Generate parameters of adaptive weights and primitive function perturbations;

Step 3: Update the sequence candidate sets using the four operation operators of AOA;

Step 4: Update the candidate solution sets by performing the adaptive weight and elementary function perturbation strategies on the sequence candidate solution set;

Step 5: Add the sequences satisfying the combination constraint to the candidate sets;

Step 6: Judge whether the maximum iteration number is reached; if so, the candidate sets satisfying the combination constraint are retained and computed; otherwise, return to Step 3.

It is assumed that $A^{GC,NL}(n,d)$ denotes the sets of DNA sequences that meet the constraints of the GC content, Hamming distance, and no-runlength, where n and d denote the sequence length and Hamming distance, respectively. The IAOA results are compared with the results obtained by the Altruistic [43] and NOL-HHO [44] algorithms proposed by Limbachiya et al. and Yin et al., respectively. Table 4 compares the IAOA results with the lower bounds of the storage sets obtained by the two comparison algorithms, satisfying the conditions 4≤n≤10 and 3≤d≤n; the best results are presented in bold. As shown in Table 4, the number of DNA coding sets can be significantly increased using the IAOA. The lower bound of the coding sets obtained by the IAOA is 25–77.7% and 5.6–23.1% higher than those of the Altruistic and NOL-HHO algorithms, respectively, when the condition of n=8 is satisfied.

In summary, by raising the lower bound of the DNA coding sets, the IAOA can ensure the storage of large amounts of data. In addition, using the IAOA, the coding rate is increased due to the increase in the number of coding sets. The coding rate [45] is defined as $R={log}_{4}^{}M/n$, where n and M denote the codeword length and coding-set size, respectively. For instance, in [44], for n=9 and d=4, $R={log}_{4}^{}216/9\approx 0.43$, and when n=8 and d=4, the coding rate obtained by the IAOA reaches 0.43, indicating that shorter sequences can achieve the same performance.

### 3.7. Encoding Sets Constructed Using Double-Matching and Error-Pairing Constraints

In this study, double-matching and error-pairing constraints were added to the traditional constraint combinations to obtain high-quality DNA storage sets. It is assumed that $A^{GC,NL,DM}(n,d)$ denotes the DNA coding sets, satisfying the constraints of the GC content, Hamming distance, no-runlength, and double-matching; then, the results of the lower bounds are presented as shown in Table 5. It is assumed that $A^{GC,NL,EP}(n,d)$ denotes the DNA coding sets, satisfying the constraints of the GC content, Hamming distance, no-runlength, and error-pairing; then, the results of lower bounds are as presented in Table 6. It is assumed that $A^{GC,NL,DM,EP}(n,d)$ denotes the DNA coding sets, satisfying the constraints of the GC content, Hamming distance, no-runlength, double-matching, and error-pairing; then, the results of lower bounds are as presented in Table 7.

### 3.8. Storage Set Quality Comparison

The melting temperature represents the temperature at which the DNA is changed from double-strand to single-strand [46]. It is an influential parameter in determining the reaction efficiency and a relatively stable melting temperature facilitates the reaction. The hairpin structure [47] is a secondary structure that is created by stacking the DNA sequence. If the hairpin structure is formed during storage, the physical structure of the sequence is unstable. The melting temperature of the DNA sequences was calculated using the Integrated DNA Technologies (IDT) platform, and then its variance and the hairpin structure were calculated by Equation (19).

(19) $$f_{hairpin}\left(X\right)=\sum_{r}^{\left(n-2\times plen\right)}\sum_{i=plen+[\frac{r}{2}]}^{\left(n-plen-[\frac{r}{2}]\right)}hairpin(X,i)$$

where *r* and *plen* stand for the smallest subsequence lengths required to form, respectively, a hairpin ring and a hairpin stem. When a hairpin structure forms at the *i*th base of a sequence, the value of hairpin(*X*, *i*) is 1; otherwise it is 0, depending on whether the number of complementary bases in the stem of a hairpin is greater than half the length of the stem.

The variance of the melting temperature and the number of hairpin structures of $A^{GC,NL}(n,d)$ and $A^{GC,NL,DM,EP}(n,d)$ are compared. The comparison results show that the quality of the DNA sequence constructed under the double-matching and error-pairing constraints is improved.

Table 8 compares the melting temperature variance of $A^{GC,NL}(n,d)$ and $A^{GC,NL,DM}(n,d)$. Table 9 compares the melting temperature variance of $A^{GC,NL}(n,d)$ and $A^{GC,NL,EP}(n,d)$. Table 10 compares the melting temperature variance of $A^{GC,NL}(n,d)$ and $A^{GC,NL,DM,EP}(n,d)$. Table 10 shows that the melting temperature variance of DNA sequences was significantly reduced after adding the double-matching and error-pairing constraints. For example, for n=8,9, and 10, the melting temperature variance of $A^{GC,NL,DM,EP}(n,d)$ reduced by 15.8–42.5%, 26.3–74.5%, and 9.7–84.1%, respectively, compared with that of $A^{GC,NL}(n,d)$, demonstrating that the DNA sequences constructed using new constraints almost all have the same melting temperature.

The comparison results of the hairpin structure number and hairpin structure ratio (i.e., the ratio of the number of hairpin structures to the number of DNA sequences) of $A^{GC,NL,DM}(n,d)$ and $A^{GC,NL,EP}(n,d)$ are shown in Table 11 and Table 12, respectively. The comparison results of the hairpin structure number and hairpin structure ratio of $A^{GC,NL}(n,d)$ and $A^{GC,NL,DM,EP}(n,d)$ are shown in Table 13 and Table 14, respectively. Table 14 shows that adding the new constraints significantly reduces the number of hairpin structures in DNA sequences. For example, for n=8,9, and 10, the ratio of the number of hairpin structures to the number of DNA sequences of $A^{GC,NL,DM,EP}(n,d)$ reduced by 2.1–50%, 4.2–23.1%, and 6.4–80%, respectively, compared to that of $A^{GC,NL}(n,d)$, demonstrating that the DNA sequences constructed using the proposed constraints can reduce the probability of hairpin structures occurring and enhance the stability of DNA sequences.

By evaluating the hairpin structure and melting temperature, it is evident that the physical and thermodynamic properties of the DNA storage sets using the double-matching and error-pairing constraints are more stable. In addition, sequences that have fewer hairpin structures can effectively avoid the occurrence of nonspecific hybridization reactions. Moreover, a stable melting temperature enhances the stability of storage in the DNA strand, which ensures a smooth hybridization reaction during DNA storage, improving the DNA sequence quality.

## 4. Conclusions

Aiming to decrease the occurrence of non-specific hybridization reactions during DNA storage and reduce the error rate of DNA storage, this paper proposed a method to improve the DNA coding set quality by introducing the double-matching and error-pairing constraints. DNA sequences may undergo complementary pairing reactions after entering the solution, thus reducing the concentration of DNA sequences and affecting the coding quality. First, a double-matching constraint was proposed to solve this problem. The comparative results of the NUPACK simulations demonstrate that this constraint can effectively increase the concentration of sequences. Further, the error-pairing constraint was proposed for the situation where the base distribution is not reasonable, thus causing mismatches, and the mismatch may trigger the synthesis of the strand and obtain nonideal amplification products. This constraint restricts the base distribution at the 3′ end and evaluates the mismatch level. In addition, this study proposed the IAOA, which adopts two main strategies, namely the elementary function random perturbation and the dual adaptive weighting strategy. To visualize the optimization performance of the IAOA, it was compared with seven other metaheuristics, including the AOA, using 13 different test functions to evaluate the IAOA. Based on the test results, the IAOA achieves the ideal value on almost all tested functions and theoretical optimal values on seven tested functions. The experimental results show that the IAOA has a more competitive performance than the other algorithms. Finally, this paper combined the IAOA with traditional constraints to construct DNA storage sets. Compared to previous studies, the lower limit of the DNA coding set obtained in this study was increased by 77.7%, indicating that the IAOA has powerful development and exploration capabilities to construct many DNA storage sets and increase the coding rate. To further test the validity of the proposed constraints and the quality of the code set, the melting temperature and hairpin structure number were evaluated and compared. The comparison results show that the melting temperature variance of the DNA sequence obtained using the new constraints was reduced by 9.7–84.1%, and the hairpin structure ratio was reduced by 2.1–80%. It was demonstrated that, compared to traditional constraints, the sequences obtained under the proposed constraints have superior physical and thermodynamic properties, maintaining a relatively stable state and reducing nonspecific hybridization.

This study introduced the double-matching and error-pairing constraints, which can effectively improve the performance of storage sets. However, as for the problem of the hairpin structure generation in DNA sequences, this problem can only be addressed to a certain extent by using the proposed constraints but cannot be completely solved. In [23], Li et al. proposed a repeat tandem sequence constraint and improved the DTW distance constraint. The repeat tandem sequence constraint was used to address the problem of the secondary structure arising from successively repeated occurrences of coding sequences, and the improved DTW distance constraint was used to address the problem of inaccurate assessment of the overall similarity between sequences obtained under the traditional distance constraint. Their proposed constraints mainly made restrictions and adjustments for a sequence as a whole, which had good advantages for constraining DNA sequences but could not restrict the local bases of DNA sequences and might not be as effective for short DNA sequences. In this paper, the proposed double-matching and error-pairing constraints are mainly used to avoid complementary reactions of sequences and restrict the bases at the 3′ ends of sequences. The focus is on solving the problem of local base appearance. This study compared the lower bound data from the coding set with the results presented in [23] and found that the results presented in [23] show a good advantage in terms of finding the lower bounds of the coding sets of long sequences. However, for the lower bounds of the coding sets of shorter sequences, the results presented in this study are better than the results in [23] and [45], and some of the data presented in the study conducted by Li et al. are inferior to those presented in [45]. Because the constraints proposed in this paper and those proposed by Li et al. [45] are different, the performance of the data in long and short sequences differs, which is also due to the different focus of algorithms, i.e., the IAOA proposed in this study and the ROEAO proposed by Li et al.

In future studies, the characteristic that the constraint in [23] has a better limiting effect on long sequences could be combined with the proposed constraints to compensate for the shortcomings of this study. In addition, the algorithm in [23] has a good global search performance, and this advantage could be used to obtain more optimal solutions. Both of the mentioned aspects are crucial for constructing a large number of high-quality coding sets. In addition, the other methods could be further explored to improve the encoding efficiency and save storage costs, in addition to constructing large codebooks.

## Figures and Tables

**Table 1:** Results of the single-peak function test with 30 dimensions.

ID	Metric	IAOA	EAOA	DAOA	pAOA	AOA	SCA	SSA	WOA	GSA	MVO
F1	AVG	**0**	8.06 × 10^−6^	**0**	7.17 × 10^−7^	8.65 × 10^−26^	7.6776	1.58 × 10^−7^	1.41 × 10^−30^	2.53 × 10^−16^	1.34 × 10^0^
	STD	**0**	2.88 × 10^−5^	**0**	1.87 × 10^−6^	4.74 × 10^−25^	12.3019	1.71 × 10^−7^	4.91 × 10^−30^	9.67 × 10^−17^	5.38 × 10^−1^
F2	AVG	**0**	9.93 × 10^−98^	**0**	7.74 × 10^−70^	**0**	0.01806	2.66293	1.06 × 10^−21^	0.055655	2.20 × 10^0^
	STD	**0**	5.44 × 10^−97^	**0**	4.24 × 10^−69^	**0**	0.02457	1.66802	2.39 × 10^−21^	0.194074	7.31 × 10^0^
F3	AVG	**0**	0.002257	**0**	0.002124	0.008014	9961.453	1709.94	5.39 × 10^−7^	896.5347	2.04 × 10^2^
	STD	**0**	0.002291	**0**	0.00177	0.01192	6699.979	11242.3	2.93 × 10^−6^	318.9559	6.63 × 10^1^
F4	AVG	**0**	0.012309	**0**	0.008175	0.02667	36.7941	11.6741	0.072581	7.35487	2.16 × 10^0^
	STD	**0**	0.004386	**0**	0.003422	0.02021	13.1414	4.1792	0.39747	1.741452	8.66 × 10^−1^
F5	AVG	**27.7041**	28.5909	28.0863	28.5339	28.3946	27188.68	296.125	27.86558	2.84 × 10^1^	7.89 × 10^2^
	STD	0.27902	0.29615	0.34232	**0.13585**	0.3301	72171.04	508.863	0.763626	2.00 × 10^−1^	8.74 × 10^2^
F6	AVG	1.937	2.4126	4.8677	1.5769	3.2316	21.998	1.80 × 10^−7^	3.116266	**2.50 × 10^−16^**	1.34 × 10^0^
	STD	0.38153	0.44494	0.50584	0.2591	0.2455	27.8352	3.00 × 10^−7^	0.532429	**1.74 × 10^−16^**	3.43 × 10^−1^
F7	AVG	5.42 × 10^−5^	6.25 × 10^−5^	7.40 × 10^−5^	**4.51 × 10^−5^**	5.45 × 10^−5^	0.08458	0.1757	0.001425	0.089441	3.21 × 10^−2^
	STD	7.85 × 10^−5^	8.45 × 10^−5^	8.20 × 10^−5^	**3.70 × 10^−5^**	5.15 × 10^−5^	0.09798	0.0629	0.001149	0.04339	1.32 × 10^−2^

**Table 2:** Results of the multipeak function test with 30 dimensions.

ID	Metric	IAOA	EAOA	DAOA	pAOA	AOA	SCA	SSA	WOA	GSA	MVO
F8	AVG	−6717.1783	−6021.7325	−4156.4559	−6953.88	−5395.427	−3771.665	−7455.8	−5080.76	−2821.07	**−7550**
	STD	580.0765	698.8845	599.9788	424.3747	436.8011	**293.4553**	772.811	695.7968	493.0375	6.27 × 10^2^
F9	AVG	**0**	**0**	**0**	**0**	**0**	41.6519	58.3708	**0**	25.96841	1.20 × 10^2^
	STD	**0**	**0**	**0**	**0**	**0**	44.3312	20.016	**0**	7.470068	3.29 × 10^1^
F10	AVG	**8.88 × 10^−16^**	**8.88 × 10^−16^**	**8.88 × 10^−16^**	**8.88 × 10^−16^**	**8.88 × 10^−16^**	14.2857	2.6796	7.4043	0.062087	2.03 × 10^0^
	STD	**0**	**0**	**0**	**0**	**0**	8.5929	0.8275	9.897572	0.23628	5.47 × 10^−1^
F11	AVG	**0**	0.004063	**0**	1.31 × 10^−5^	0.1689	0.8665	0.016	0.000289	27.70154	8.60 × 10^−1^
	STD	**0**	0.009628	**0**	8.60 × 10^−6^	0.1347	0.3957	0.0112	0.001586	5.040343	8.21 × 10^−2^
F12	AVG	**0.20433**	0.5004	0.70028	0.2241	0.5195	183961.2	6.9915	0.339676	1.799617	2.43 × 10^0^
	STD	**0.034199**	0.043832	0.066711	0.0416	0.04741	841708.7	4.4175	0.214864	0.95114	1.39 × 10^0^
F13	AVG	2.2663	2.7269	2.7643	2.7669	2.8475	109173.3	15.8757	1.889015	8.899084	**1.96 × 10^−1^**
	STD	0.24611	0.30181	0.298724	0.1274	**0.07852**	266184.2	16.1462	0.266088	7.126241	1.26 × 10^−1^

**Table 3:** Wilcoxon statistical test result.

Comparison	*p*-Value
IAOA-AOA	0.005847
IAOA-pAOA	0.028056
IAOA-SCA	0.001474
IAOA-SSA	0.039243
IAOA-WOA	0.026231
IAOA-GSA	0.009633
IAOA-MVO	0.008775

**Table 4:** Lower bounds of $A^{GC,NL}(n,d)$, where the best results are bolded to indicate.

n\d		3	4	5	6	7	8	9
	Altruistic	11						
4	NOL-HHO	**12**						
	IAOA	**12**						
	Altruistic	17	7					
5	NOL-HHO	**20**	**8**					
	IAOA	**20**	**8**					
	Altruistic	44	16	6				
6	NOL-HHO	55	23	**8**				
	IAOA	**61**	**24**	**8**				
	Altruistic	110	36	11	4			
7	NOL-HHO	121	42	14	**7**			
	IAOA	**136**	**46**	**16**	**7**			
	Altruistic	289	86	29	9	4		
8	NOL-HHO	339	108	35	13	**5**		
	IAOA	**373**	**114**	**39**	**16**	**5**		
	Altruistic	662	199	59	15	8	4	
9	NOL-HHO	705	216	69	22	**11**	4	
	IAOA	**789**	**231**	**71**	**27**	**11**	**5**	
	Altruistic	1810	525	141	43	7	5	4
10	NOL-HHO	1796	546	148	51	20	9	4
	IAOA	**1945**	**549**	**156**	**56**	**22**	**10**	**5**

**Table 5:** Lower bounds for $A^{GC,NL,DM}(n,d)$.

n\d	3	4	5	6	7	8	9
4	12						
5	20	8					
6	58	22	8				
7	125	42	17	6			
8	322	96	29	13	5		
9	587	194	50	18	9	6	
10	1206	398	117	46	16	8	4

**Table 6:** Lower bounds for $A^{GC,NL,EP}(n,d)$.

n\d	3	4	5	6	7	8	9
4	12						
5	20	8					
6	60	23	8				
7	126	43	18	6			
8	338	119	33	14	5		
9	598	201	58	21	11	7	
10	1391	408	126	49	18	8	4

**Table 7:** Lower bounds for $A^{GC,NL,DM,EP}(n,d)$.

n\d	3	4	5	6	7	8	9
4	12						
5	20	8					
6	45	17	7				
7	124	42	15	6			
8	245	79	28	11	5		
9	577	178	54	19	9	4	
10	1073	374	110	39	15	8	4

**Table 8:** Comparison results of the melting temperature variance, where the best results are bolded to indicate.

n\d		3	4	5	6	7	8	9
8	$A^{GC,NL}$	3.5538	4.0477	6.0929	5.4760	4.6825		
	$A^{GC,NL,DM}$	**3.0968**	**3.3164**	**5.3873**	**5.1429**	**4.3069**		
9	$A^{GC,NL}$	3.3585	2.6020	5.6688	4.6172	4.5196	4.8333	
	$A^{GC,NL,DM}$	**2.7969**	**2.5061**	**4.3512**	**4.1070**	**4.2980**	**4.7471**	
10	$A^{GC,NL}$	6.8984	2.9266	3.3843	3.2426	4.3286	2.6257	3.0167
	$A^{GC,NL,DM}$	**5.9291**	**2.7699**	**2.7484**	**2.8492**	**3.2602**	**2.5596**	**2.8941**

**Table 9:** Comparison results of the melting temperature variance, where the best results are bolded to indicate.

n\d		3	4	5	6	7	8	9
8	$A^{GC,NL}$	3.5538	4.0477	6.0929	5.4760	4.6825		
	$A^{GC,NL,EP}$	**3.0775**	**3.2602**	**5.1959**	**4.7633**	**3.9234**		
9	$A^{GC,NL}$	3.3585	2.6020	5.6688	4.6172	4.5196	4.8333	
	$A^{GC,NL,EP}$	**2.7502**	**1.9650**	**4.0618**	**3.6314**	**3.9391**	**4.0712**	
10	$A^{GC,NL}$	6.8984	2.9266	3.3843	3.2426	4.3286	2.6257	3.0167
	$A^{GC,NL,EP}$	**5.1916**	**2.7484**	**2.4791**	**2.7502**	**3.3756**	**2.3892**	**2.8166**

**Table 10:** Comparison results of the melting temperature variance, where the best results are bolded to indicate.

n\d		3	4	5	6	7	8	9
8	$A^{GC,NL}$	3.5538	4.0477	6.0929	5.4760	4.6825		
	$A^{GC,NL,DM,EP}$	**2.6499**	**3.4963**	**4.7498**	**4.4845**	**3.2870**		
9	$A^{GC,NL}$	3.3585	2.6020	5.6688	4.6172	4.5196	4.8333	
	$A^{GC,NL,DM,EP}$	**2.5067**	**1.4909**	**3.9142**	**3.1942**	**3.5079**	**3.8270**	
10	$A^{GC,NL}$	6.8984	2.9266	3.3843	3.2426	4.3286	2.6257	3.0167
	$A^{GC,NL,DM,EP}$	**4.0036**	**2.2510**	**1.8384**	**2.5098**	**3.0063**	**2.2795**	**2.7492**

**Table 11:** Comparison results for the hairpin structure number.

n\d		3	4	5	6	7	8	9
8	$A^{GC,NL,DM}$	134	38	14	5	2		
	$A^{GC,NL,EP}$	141	48	16	6	2		
9	$A^{GC,NL,DM}$	751	251	68	25	13	7	
	$A^{GC,NL,EP}$	776	258	81	29	16	8	
10	$A^{GC,NL,DM}$	3352	1064	335	128	46	19	18
	$A^{GC,NL,EP}$	3832	1089	367	137	57	20	8

**Table 12:** Comparison results for the hairpin structure ratio.

n\d		3	4	5	6	7	8	9
8	$A^{GC,NL,DM}$	0.4162	0.3958	0.4828	0.3846	0.4		
	$A^{GC,NL,EP}$	0.4172	0.4034	0.4848	0.4286	0.4		
9	$A^{GC,NL,DM}$	1.2794	1.2938	1.36	1.3889	1.4444	1.1667	
	$A^{GC,NL,EP}$	1.2977	1.2835	1.3966	1.3810	1.4545	1.1429	
10	$A^{GC,NL,DM}$	2.7794	2.6734	2.8632	2.7826	2.875	2.375	2.25
	$A^{GC,NL,EP}$	2.7549	2.6691	2.9127	2.7959	3.1667	2.5	2.75

**Table 13:** Comparison results for the hairpin structure number, where the best results are bolded to indicate.

n\d		3	4	5	6	7	8	9
8	$A^{GC,NL}$	156	47	19	8	3		
	$A^{GC,NL,DM,EP}$	**127**	**37**	**15**	**4**	**2**		
9	$A^{GC,NL}$	1024	308	100	38	17	6	
	$A^{GC,NL,DM,EP}$	**683**	**222**	**73**	**24**	**12**	**4**	
10	$A^{GC,NL}$	5412	1482	460	158	76	25	18
	$A^{GC,NL,DM,EP}$	**2815**	**1033**	**327**	**125**	**40**	**17**	**8**

**Table 14:** Comparison results for the hairpin structure ratio, where the best results are bolded to indicate.

n\d		3	4	5	6	7	8	9
8	$A^{GC,NL}$	0.4182	0.4123	0.4872	0.5	0.6		
	$A^{GC,NL,DM,EP}$	**0.4097**	**0.3895**	**0.4688**	**0.3636**	**0.4**		
9	$A^{GC,NL}$	1.2978	1.3333	1.4085	1.4074	1.5455	1.2	
	$A^{GC,NL,DM,EP}$	**1.1837**	**1.2472**	**1.3519**	**1.1429**	**1.3333**	**1**	
10	$A^{GC,NL}$	2.7825	2.6995	2.9487	2.8214	3.4545	2.5	3.6
	$A^{GC,NL,DM,EP}$	**2.3576**	**2.4654**	**2.7712**	**2.7778**	**2.5**	**2.125**	**2**

## Data Availability

The data that support the findings of this study are available from UCI Machine Learning Repository. Restrictions apply to the availability of these data, which were used under license for this study.
